# A Differentiation-Based Phylogeny of Cancer Subtypes

**DOI:** 10.1371/journal.pcbi.1000777

**Published:** 2010-05-06

**Authors:** Markus Riester, Camille Stephan-Otto Attolini, Robert J. Downey, Samuel Singer, Franziska Michor

**Affiliations:** 1 Computational Biology Center, Memorial Sloan-Kettering Cancer Center, New York, New York, United States of America; 2 Department of Bioinformatics, University of Leipzig, Leipzig, Germany; 3 Department of Surgery, Memorial Sloan-Kettering Cancer Center, New York, New York, United States of America; University of Washington, United States of America

## Abstract

Histopathological classification of human tumors relies in part on the degree of differentiation of the tumor sample. To date, there is no objective systematic method to categorize tumor subtypes by maturation. In this paper, we introduce a novel computational algorithm to rank tumor subtypes according to the dissimilarity of their gene expression from that of stem cells and fully differentiated tissue, and thereby construct a phylogenetic tree of cancer. We validate our methodology with expression data of leukemia, breast cancer and liposarcoma subtypes and then apply it to a broader group of sarcomas. This ranking of tumor subtypes resulting from the application of our methodology allows the identification of genes correlated with differentiation and may help to identify novel therapeutic targets. Our algorithm represents the first phylogeny-based tool to analyze the differentiation status of human tumors.

## Author Summary

Gene expression profiling of malignancies is often held to demonstrate genes that are “up-regulated” or “down-regulated”, but the appropriate frame of reference against which observations should be compared has not been determined. Fully differentiated somatic cells arise from stem cells, with changes in gene expression that can be experimentally determined. If cancers arise as the result of an abruption of the differentiation process, then poorly differentiated cancers would have a gene expression more similar to stem cells than to normal differentiated tissue, and well differentiated cancers would have a gene expression more similar to fully differentiated cells than to stem cells. In this paper, we describe a novel computational algorithm that allows orientation of cancer gene expression between the poles of the gene expression of stem cells and of fully differentiated tissue. Our methodology allows the construction of a multi-branched phylogeny of human malignancies and can be used to identify genes related to differentiation as well as novel therapeutic targets.

## Introduction

Cancer research has traditionally focused on the identification of oncogenes and tumor suppressor genes, but in the last decades it has become increasingly apparent that disruption of normal differentiation is an important component of tumorigenesis. Lack of cellular maturation is now recognized as a hallmark of human cancers [1], and the degree of differentiation of a tumor is important for diagnosis, prognosis, and treatment. Investigations of hematopoietic malignancies, for instance, have benefited considerably from an understanding of the differentiation hierarchy of hematopoietic cells. The identification of immunophenotypic markers and gene expression profiles correlated with maturation has enabled researchers to map the expansion of malignant cells to particular stages of hematopoietic differentiation [2]. Such characterization has proven invaluable for diagnostic and prognostic purposes, and continues to provide clues for pharmacological interventions. Furthermore, the extent of differentiation indicated by the histologic subtype of liposarcoma is the most important determinant of the clinical outcome for this cancer type [3]–[5]. Nevertheless, attempts to categorize solid tumors have proven difficult due to an incomplete understanding of differentiation pathways from stem cells into mesenchymal and epithelial tissues. The classifications undertaken so far have been based on *in vitro* measurements of genes expressed during the differentiation of stem cells into mature tissue; this data was then compared to expression profiles of different tumor subtypes to identify the maturation stages to which these subtypes correspond [6]. However, such approaches are not yet widely applicable since the prospective isolation of tissue-specific stem cells has been possible for only few tissue types, e.g. hematopoietic, mesenchymal, epithelial, and neural tissues ([7] and references therein). Similarly, *in vitro* methods of differentiation are available for only a few histologies [8]. Furthermore, the necessity of an array of growth factors for *in vitro* differentiation raises questions about the similarity of the *in vitro* model to *in vivo* processes. Often only a fraction of cells undergoes differentiation under *in vitro* conditions, and currently available methods do not allow isolation of those cells during the differentiation process from the bulk of unchanged cells.

An objective categorization of cancers according to maturity requires a methodology that does not depend on expression data obtained from *in vitro* models of differentiation. In this paper, we develop a novel computational algorithm that assigns a degree of dissimilarity from stem cells to human cancer subtypes. Our methodology utilizes gene expression data of tumor subtypes to construct a phylogenetic tree based on genes differentially expressed among the subtypes, as well as gene expression data of stem cells and fully differentiated cells. The resulting phylogeny provides information about the maturation status of tumor subtypes and the relationship between them. The results of our algorithm are conceptually similar to the mapping of cellular expansion occurring during hematopoietic malignancies to the differentiation hierarchy of hematopoiesis. Our methodology allows classification of cancer subtypes according to their maturation status, to identify genes whose expression correlates with differentiation, and to discover candidate genes which are promising therapeutic targets. Our methodology is part of an increasing literature of mathematical and statistical investigations of cancer [9]–[14].

## Results

### Phylogenetic tree reconstruction method

**Figure 1:**
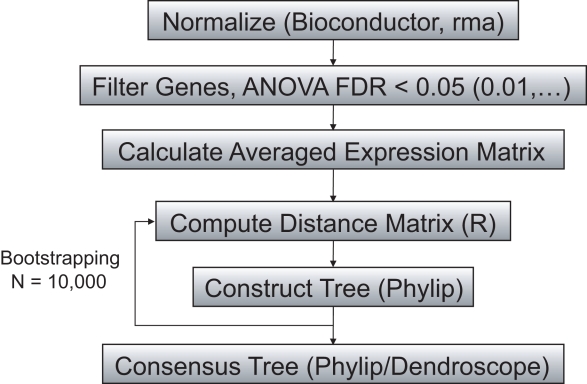
Schematic outline of the methodology. The flow chart shows the main steps of the algorithm used to construct a phylogenetic tree of tumor subtypes. First, the data is normalized using the *Bioconductor* software. Then ANOVA is used to identify those genes that are differentially expressed in at least one tumor subtype; we use a False Discovery Rate (FDR) of less than 0.01. Afterwards, the expression of each differentially expressed gene is averaged across all samples of each subtype. Those average expression levels are then used to compute the distance matrix of the subtypes, which is in turn utilized to construct a phylogenetic tree using the *Phylip* or *FastME* software. To determine the consensus tree, the phylogenetic construction is repeated 10,000 times using different sets of differentially expressed genes (of varying number). The consensus tree produced with this bootstrapping approach is visualized with the *Dendroscope* software.

Our algorithm uses gene expression data of tumor samples that have been pathologically classified into subtypes. The expression data is normalized and then analyzed for differentially expressed genes, i.e. those genes whose expression in samples from one tumor subtype differs from the expression in samples from at least one other subtype. We use these genes to compute the distances between all pairs of subtypes; the resulting distance matrix is then used to construct a phylogenetic tree. This construction is repeated several thousand times using different subsets of genes (of varying size) to estimate the statistical significance of the branches of the tree (Fig. 1). We perform a systematic analysis of several methods and parameters used in our algorithm (see Methods for details). We find that combining ANOVA and Benjamini-Hochberg with a p-value of 0.01 gives good and robust results, while the Weighted Least Squares (WLS) tree reconstruction method works best when combined with the Pearson correlation matrix. Other combinations of methods give similar results and therefore should be tested in order to have an accurate understanding of a given dataset.

The phylogenetic tree resulting from this analysis contains information about the relation among subtypes as well as between subtypes and the root of the tree. The branching points represent the ‘common ancestors’ of the subtypes that are situated at the leaves of those branches. If the tree is rooted with expression data of a primitive cell type such as embryonic or tissue-specific stem cells, then the subtypes that are located more closely to the root correspond to types that are more similar to stem cells while the subtypes that are located farthest away from the root represent the most dissimilar types. The order of the branching points along the differentiation course can be interpreted as the ranking in dissimilarity of each of the subtypes to stem cells. The differences between stem cells and tumor subtypes are in part caused by different differentiation status and in part by the abnormal cancer phenotype. In some situations, the order of the subtypes dictated by the tree is not unique, resulting from a non-fully balanced tree. For instance, more than one subtype can be mapped to exactly the same point in the ordering according to dissimilarity from stem cells. Furthermore, the two subtypes farthest away from the root share the same common ancestor and therefore cannot be distinguished in their level of dissimilarity. To resolve this conflict, expression data of a fully differentiated cell type can be included, which unambiguously defines the last branching point in the ranking.

We validate our methodology with three datasets: (i) a dataset containing gene expression data of acute myeloid leukemia (AML) samples which are categorized according to the French-American-British (FAB) classification into classes that mirror maturation status [2]; (ii) a dataset containing gene expression of breast cancer samples classified according to estrogen receptor status and Elston histological grade [15]–[17]; and (iii) a dataset containing gene expression data of liposarcoma subtypes which have been analyzed for their differentiation status by comparing them to an *in vitro* differentiation time course [6].

Acute myeloid leukemia (AML) is a clonal disease characterized by the accumulation of myeloid progenitor cells in blood and bone marrow [18]. AML results from changes in transcription factor regulation that lead to a disruption of normal cellular differentiation. AML is classified into seven distinct subtypes depending on the morphology and differentiation status of tumor cells: dedifferentiated, myeloblastic, myeloblastic with maturation, promyelocytic, myelomonocytic, monocytic, and erythroleukemic AML. According to the FAB classification, these subtypes are denoted by M0, M1, …, and M6, respectively. Since AML is the result of alterations of the differentiation process, we validate our approach with a dataset of gene expression of AML patients.

Our leukemia dataset contains gene expression data of 362 AML patients and of 7 patients with unclassified Myelodysplastic Syndrome (MDS) (see Methods for details of dataset compilation) (Table 1). To root the AML tree, we use expression data of human embryonic stem cells (hESC); additionally, we include expression data of CD34+ hematopoietic cells from both peripheral blood (CD34 PB) and bone marrow (CD34 BM), human mesenchymal precursor cells (hESC MPC), as well as fully differentiated mononuclear cells from peripheral blood (PB) and bone marrow (BM). The surface glycophosphoprotein CD34 is expressed on undifferentiated hematopoietic stem and progenitor cells [19] and is widely used as a marker for less differentiated hematopoietic cells. We include these two subgroups as a further test of our methodology since their differentiation status is known. We use ANOVA to identify those probe sets that are significantly differentially expressed in at least one subtype as compared to all other AML subtypes. The analysis identifies 11,105 probe sets that are differentially expressed among AML subtypes if a false discovery rate (FDR) [20] of 0.01 is used. Use of this cutoff would lead us to expect 111 false positives. If we use the Holm correction method instead [21], which controls the family-wise error rate, then the number of differentially expressed probe sets decreases to 4,051 (with 0.01 expected false positives). The inclusion of less significantly differentially expressed genes is a potential source of noise; however, high cutoffs for significance discard genes that could be interesting for further analysis. The tradeoff between these two effects must be examined carefully to choose an appropriate cutoff. We decided to use a standard cutoff FDR of 0.01 because the tree topology remains stable for large gene sets, and also a larger number of potentially interesting genes are included which can be further filtered with other techniques.

**Table 1:** French-American-British (FAB) classification of acute myeloid leukemia (AML) subtypes and numbers of samples.

FAB class	Name of subtype	Number of samples
M0	Dedifferentiated	14
M1	Myeloblastic	78
M2	Myeloblastic with maturation	78
M3	Promyelocytic	29
M4	Myelomonocytic	75
M5	Monocytic	78
M6	Erythroleukemic	10

**Figure 2:**
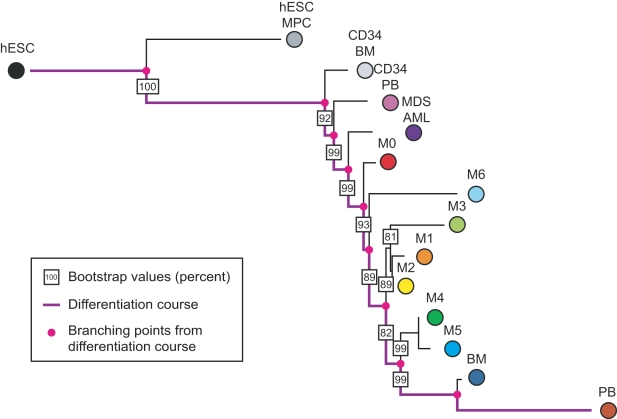
A phylogeny of acute myeloid leukemia (AML) subtypes. According to the French-American-British (FAB) classification, AML samples are classified into seven different types according to their level of differentiation (see Table 1). Expression data from 362 AML patients and 7 Myelodysplastic Syndrome (MDS-AML) patients is used to construct a phylogeny of these leukemias. We include expression data of human embryonic stem cells (hESCs), CD34+ cells from bone marrow (CD34 BM) and peripheral blood (CD34 PB), and mononuclear cells from bone marrow (BM) and peripheral blood (PB). The differentiation pathway from hESCs to mononuclear cells from peripheral blood is represented in purple, and the common ancestors of subtypes are shown as pink dots. The bootstrap values of branches are indicated by boxed numbers, representing the percentage of bootstrapping trees containing this branch. The ranking of AML subtypes identified by the phylogenetic algorithm corresponds with the differentiation status indicated by the FAB classification. The M6 subtype, represented by only 10 samples in our dataset, has the least stable branch, leading to lower bootstrap values for those branches where it can alternatively be located.

The table shows the names of subtypes as classified by FAB and the numbers of samples included in our study (see Fig. 2).

The consensus phylogenetic tree based on this data is shown in Fig. 2. The order of the branching points of the subtypes coincides with the differentiation stages specified by the FAB classification: dedifferentiated AML (the M0 subtype) is located close to the stem cells while myelomonocytic (M4) and monocytic (M5) AML are located in the most distant leaves of the tree. The inner branching of the tree is also in accordance with the differentiation status suggested by the FAB classification (Table 1). The tree topology specifying the correct order of myeloblastic and promyelocytic maturation (M2 and M3), however, only has a moderate bootstrap value because the two subtypes are very similar in maturity. The branch leading to the erythroleukemic subtype (M6) is relatively unstable. This could be attributed to the small number of samples in this subtype or to a possible misclassification or erroneous diagnosis. Therefore, the position of this subtype in the tree is less certain than that of other subtypes; this uncertainty decreases the bootstrap values of the other branches at which this subtype can be located. All other branches in the tree are very stable under bootstrapping. Of central importance for the interpretation of the results is how well the tree captures the observed relationships in the data. A good measure of this fit is the average percent standard deviation of the distances between subtypes in the data compared to the ones in the tree. The Least Squares algorithm minimizes this score. For the Pearson correlation distance, the mean observed average percent deviation is 12.05%, which is a reasonable fit for this distance measure [22]; hence our algorithm produces a phylogeny which accurately recapitulates the relationships seen in the data.

**Figure 3:**
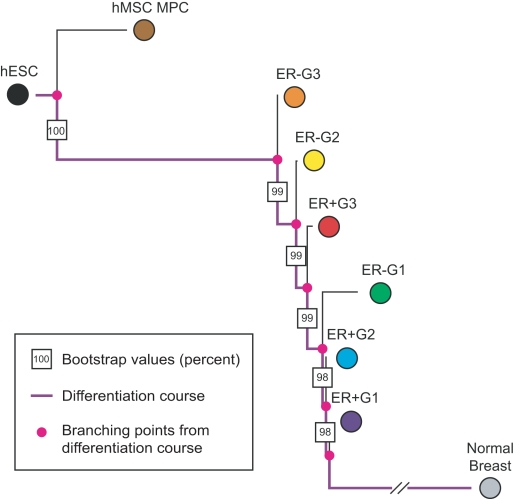
A phylogeny of breast cancer subgroups. The figure shows the consensus tree of breast cancer subgroups. We use expression data of 483 breast cancer samples subdivided as shown in Table 2. The tree is rooted with expression data of human mesenchymal stem cells (hMSCs). We also include expression data of fully differentiated normal breast tissue. The differentiation pathway from hESC to fully differentiated breast tissue is indicated in purple, and the pink dots represent the common ancestors of (sets of) subgroups. The boxed numbers specify the bootstrap values of branches. The phylogeny ranks the breast cancer subtypes according to their dissimilarity from stem cells as ER− grade 3, ER− grade 2, ER+ grade 3, followed by ER− grade 1, ER+ grade 2 and ER+ grade 1.

We also apply our algorithm to a breast cancer dataset in order to study the performance of our method using cancers of epithelial origin. The samples in our dataset were characterized by immunochemistry methods according to their estrogen receptor status (ER+ and ER−) and Elston histologic grade (G1, G2, and G3). We compile a total of 483 unique samples, among which we find all combinations of ER status and grade (Table 2). The raw data is analyzed as described in the methods section. We root the tree with human mesenchymal stem cells and also include samples of normal breast [23]. Results are shown in Fig. 3. We find 17,966 probes differentially expressed between the subgroups when using ANOVA with Benjamini-Hochberg correction and a cutoff value of 0.01. A negative ER status has been shown to correlate with poor prognosis [24]. Consistent with this observation, our algorithm places ER-negative subgroups closer to stem cells, reflecting the more stem-like properties of these aggressive tumors, while ER+ tumors are placed closer to the normal breast tissue samples. Tumor grades are ordered similarly, placing tumors of higher grade closer to stem cells. Most trees reconstructed with the different sets of genes have the same topology (bootstrap values close to 100%), reflecting a very robust phylogeny. We conclude that our methodology is also able to accurately rank tumors of epithelial origin according to maturity.

**Table 2:** Breast cancer subgroups and numbers of samples.

Characterization of subgroup	Number of samples
Normal breast tissue (NB CA)	14
ER − Grade 3	76
ER − Grade 2	27
ER − Grade 1	3
ER + Grade 3	84
ER + Grade 2	179
ER + Grade 1	114

The table shows the names of the subgroups contained in the breast cancer dataset and the numbers of cancer samples as well as healthy tissue samples included in our study (see Fig. 3).

Next we construct a phylogeny of liposarcoma subtypes. Liposarcoma is the most common type of soft tissue sarcoma accounting for about 20% of all tissue sarcomas [25]. In 2008, 10,390 new cases of sarcoma were reported in the US [26]. Surgery is the standard care for localized tumors but leads to worse prognoses in cases of locally advanced or disseminated disease [27]. Liposarcomas are classified into three biological types encompassing five subtypes: (i) well-differentiated/dedifferentiated, (ii) myxoid or round cell, and (iii) pleomorphic liposarcoma, based on morphological features and cytogenetic aberrations [28]. Although the subtype is the main determinant of clinical outcome [3], [4], [29]–[31], liposarcomas of similar morphology can differ in response to treatment and in prognosis [27]. Microscopically well-differentiated liposarcoma is composed of relatively mature adipocytic proliferation showing significant variation in cell size and at least focal nuclear atypia. Histologically dedifferentiated liposarcoma is represented by the transition from well-differentiated liposarcoma to non-lipogenic sarcoma. Both well-differentiated and dedifferentiated liposarcomas contain characteristic ring or giant marker chromosomes with 12q14-15 amplification. Myxoid liposarcomas contain uniform round to oval shaped primitive non-lipogenic mesenchymal cells and a variable number of small signet-ring lipoblasts in a prominent myxoid stroma. Round cell tumors are characterized by solid sheets of primitive round cells with no intervening myxoid stroma. Pleomorphic liposacoma is a pleomorphic high grade sarcoma containing a variable number of pleomorphic lipoblasts.

Recently, progress has been made towards a classification of liposarcoma subtypes utilizing gene expression data. In 2007, a 142-gene predictor was identified which correctly distinguishes between liposarcoma subtypes and generates a set of differentiation-related genes that may contain candidate therapeutic targets [27]. In 2008, Matushansky et al. showed that the main liposarcoma subtypes can be ranked according to their differentiation status by comparing gene expression data of the tumor subtypes with the genes expressed during normal *in vitro* adipogenic differentiation [6]. The ranking generated by the latter approach is useful for validating our methodology.

**Figure 4:**
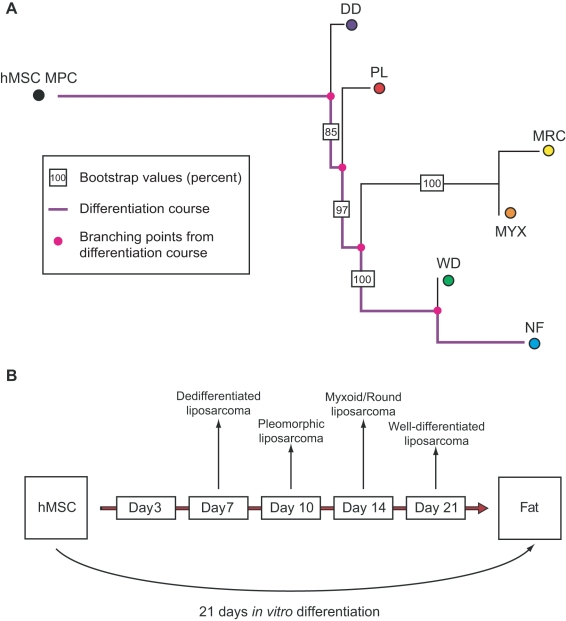
A phylogeny of liposarcoma subtypes. (**a**) The figure shows the consensus tree of liposarcoma subtypes. The tree is rooted with expression data of human mesenchymal stem cells (hMSC), and expression data of normal fat cells is included as well. The differentiation pathway from hMSC to normal fat cells is represented in purple. The pink points represent common ancestors of (sets of) subtypes. The boxed numbers specify bootstrap values of branches. The tree indicates that dedifferentiated liposarcoma is most similar to stem cells, followed by pleomorphic, myxoid, round-cell, and finally well-differentiated liposarcoma. (**b**) The figure shows a schematic representation of the correlation of adipogenesis to liposarcoma differentiation. In [6], human mesenchymal stem cells were differentiated *in vitro* to produce fat cells, and gene expression was measured for five different time points during the differentiation. The expression data of four different liposarcoma subtypes was then compared to the data obtained from the differentiation time course. This comparison identified dedifferentiated liposarcoma as the subtype most similar to stem cells, followed by pleomorphic, myxoid/round-cell, and well-differentiated liposarcoma. The correspondence between the results of our algorithm applied to gene expression datasets and these experimentally derived results serves as a validation of our methodology. Adapted from [6].

Our liposarcoma dataset includes 180 surgical samples that have been pathologically classified as 61 dedifferentiated, 52 well differentiated, 26 pleomorphic, 18 round cell, and 23 myxoid liposarcomas [27], [30]. Samples that were likely misclassified were filtered in previous studies, which is a pre-processing step critical for the outcome of the algorithm. For an FDR of the ANOVA filter of 0.01 after correction with the Benjamini-Hochberg method, we find 13,429 probe sets that are differentially expressed among the liposarcoma subtypes. Those sets are then used to construct an unrooted phylogenetic tree. To root the tree, we use expression data of mesenchymal stem cells and fully differentiated adipocytes. The resulting consensus tree is shown in Fig. 4a. The tree topology is stable with bootstrap values larger than 85%. Based on the consensus tree, the subtypes can be ordered by increasing dissimilarity from stem cells as dedifferentiated, pleomorphic, myxoid/round-cell, and well-differentiated liposarcoma (Fig. 4a). This order coincides with experimental results based on the gene expression observed during *in vitro* differentiation published earlier (Fig. 4b) [6]. By setting the p-value threshold of the Holm correction to 0.01, we obtain 7,290 differentially expressed probe sets; these probe sets generate a tree topology that is identical to the case described above with bootstrap values larger than 91.5% (data not shown). When rooting with embryonic stem cells, the branching between embryonic stem cells and the rest of the tree is less stable since the expression of embryonic stem cells differs considerably from all other samples (data not shown). To increase the stability of the tree, it is preferable to root with an outgroup that is relatively closely related to the investigated samples (in this case, mesenchymal stem cells; see also the section “Systematic analysis of methods and parameters”) [32]. Again we test how well the tree fits the distance matrix and observe a mean average percent standard deviation of 11.3%, which has been reported to be a good fit for the Pearson correlation distance [22]. Therefore, our methodology is also able to rank liposarcoma subtypes in the correct order according to their dissimilarity to stem cells.

**Figure 5:**
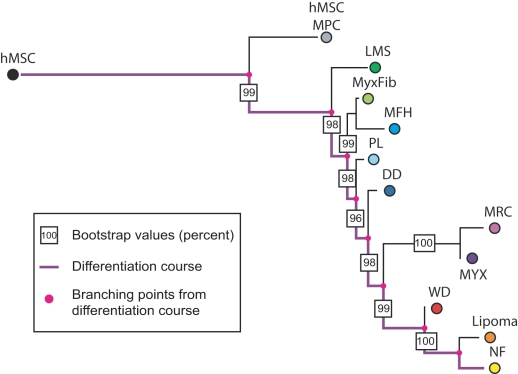
A phylogeny of sarcoma subtypes. The figure shows the consensus tree of sarcoma subtypes. We use expression data of 251 sarcoma samples classified into the types shown in Table 3. The tree is rooted with expression data of human embryonic stem cells (hESCs). We also include expression data of human mesenchymal stem cells (hMSC) and of fully differentiated normal adipocytes. The differentiation pathway from hESC to fully differentiated adipocytes is indicated in purple, and the pink dots represent the common ancestors of (sets of) subtypes. The boxed numbers specify the bootstrap values of branches. The phylogeny ranks the sarcoma subtypes according to their dissimilarity from stem cells as leiomyosarcoma, malignant fibrous histiocytoma, myxofibrosarcoma, followed by the liposarcoma subtypes dedifferentiated liposarcoma, pleomorphic, myxoid/round-cell, and well-differentiated liposarcoma. Lipoma is identified as the subtype most dissimilar from stem cells.

Since our methodology correctly ranks leukemia, breast cancer, and liposarcoma samples according to their differentiation status, we now investigate a larger number of sarcoma subtypes to identify their relationship in maturity as well as candidate targets for therapeutic intervention. The sarcoma dataset includes the 180 liposarcomas discussed above as well as 36 myxofibrosarcomas, 5 pleomorphic malignant fibrous histiocytomas (MFH), 7 lipomas, and 23 leiomyosarcomas (Table 3) [27], [30]. We use expression data of both mesenchymal stem cells and embryonic stem cells to root the tree. The consensus tree is shown in Fig. 5. Our methodology determines that leiomyosarcoma is closest in its differentiation status to stem cells, followed by MFH and myxofibrosarcoma, and finally the liposarcoma subtypes (ranked as determined above) and the benign subtype lipoma. The algorithm also clusters the subtypes according to tissue of origin, predicting that leiomyosarcoma branches before all other subtypes, and that MFH and myxofibrosarcoma have a common ancestor; so do all liposarcoma subtypes and lipoma. Note that although pleomorphic liposarcomas and MFH/myxofibrosarcomas are very similar subtypes at the level of their genetic copy number aberrations [30], our algorithm places them in different branches of the tree. This effect is a result of the phenotype-based nature of our method and is in accordance with the different tissues of origin of these subtypes. The tree has a very stable topology with bootstrap values larger than 0.90 except for the MFH subtype, which exhibits a lower bootstrap value of 0.60; this value is likely due to the small number of samples (5) available for this subtype. Note that with the current dataset, we cannot distinguish between the case in which the subtype located most closely to stem cells, leiomyosarcoma, is situated on the adipocytic differentiation path and the case in which leiomyosarcoma is alternatively located on a branch leading to fully differentiated tissue of another type. To resolve this ambiguity, gene expression data of fully differentiated tissue of all the types giving rise to sarcomas is needed.

**Table 3:** Sarcoma subtypes.

Tissue	Name of subtype	Number of samples
Fat	Dedifferentiated	61
	Pleomorphic	26
	Round-cell	18
	Myxoid	23
	Well-differentiated	52
	Lipoma	7
Smooth Muscle	Leiomyosarcoma	23
Fibrous Tissue	MFH	5
	Myxofibrosarcoma	36

The table shows the number of sarcoma subtypes included in our study (see Fig. 5).

**Figure 6:**
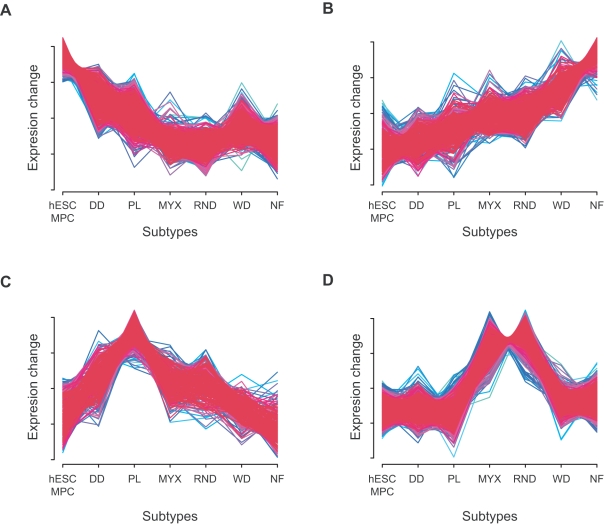
Clusters of gene expression profiles. The figure shows four example groups of differentially expressed genes clustered according to their expression profiles (see Methods section for details on the clustering algorithm). On the horizontal axis, we show the liposarcoma subtypes ordered according to the ranking identified by the phylogenetic approach (see Fig. 4a) and in the vertical axis the corresponding standard normalized average expression values of the subtypes. We also include human embryonic stem cells (hESCs) and normal fat cells. The expression of some genes continuously decreases from less differentiated samples (hESC, dedifferentiated liposarcoma, …) to more differentiated samples (…, well-differentiated liposarcoma, normal fat) (**a**), while the expression of other genes increases (**b**). Other genes are overexpressed in just a single liposarcoma subtype (**c**) or in a subset of subtypes (**d**). Those genes whose expression continuously increases or decreases are hypothesized to be related to adipogenesis (see Table 4).

We are interested in identifying genes that are related to adipogenesis, i.e. those genes that correlate with adipocyte differentiation. To identify such genes, we cluster our list of differentially expressed genes into a chosen number of groups depending on their expression pattern in sarcoma subtypes. When the subtypes are arranged according to their distance from stem cells (as indicated by the tree in Fig. 4a), the expression of some genes continuously increases from the less differentiated to the more differentiated subtypes, while the expression of other genes decreases or exhibits more complicated patterns (Fig. 6). We hypothesize that genes whose expression continuously increases or decreases are possibly related to gain of the features of differentiation and loss of stem cell-associated functions, even though this association with maturation may not be causative. To test this hypothesis, we compare the genes whose expression increases or decreases along the order of subtypes to previously published lists of adipocytic differentiation-specific genes [6], [31]. In these two studies, mesenchymal stem cells were differentiated *in vitro* into normal fat cells, and the expression profiles of cells were measured at multiple time points during the differentiation process. An investigation of genes whose expression levels changed statistically significantly along the differentiation time course led to the identification of 67 and 69 genes, respectively [6], [31]. These genes are thought to be related to adipocytic differentiation.

We rank the genes whose expression increases or decreases along the liposarcoma subtypes (see Fig. 6 for example clusters) according to the fold change between their expression in hMSC and in normal fat. Among the 11,105 probe sets obtained by the ANOVA filtering with FDR of 0.01 after Benjamini Hochberg correction, the top 25 genes in this ranking are listed in Table 4. About 64% of these genes coincide with the published lists [6], [31]. These results suggest that our methodology is able to identify differentiation-related genes from the large number of differentially expressed genes. Additionally to the previously identified genes, our method identified other genes that have not been associated with adipocytic differentiation (Table 4). For instance, the protein phosphatase inhibitor 1 (PPP1R1A) is thought to be important in the control of glycogen metabolism and is primarily expressed in liver cells; the tyrosine kinase NTRK2 is part of a signaling pathway leading to neuronal differentiation, and the metabolism related enzyme system ACACB is exclusively expressed in adipocyte tissue.

**Table 4:** Adipogenesis-related genes.

Gene Symbol	Gene Name	Fold Change (RMA Log-Ratio)
FABP4^ab^	fatty acid binding protein 4, adipocyte	352.1 (8.46)
LPL^ab^	lipoprotein lipase	164.3 (7.36)
ADH1B^ab^	alcohol dehydrogenase 1B (class I), beta polypeptide	150.1 (7.23)
HBA/B	hemoglobin	147.0 (7.20)
ADIPOQ^a^	adiponectin, C1Q and collagen domain containing	137.2 (7.14)
RBP4^ab^	retinol binding protein 4, plasma	104.0 (6.70)
GOS2^b^	G0/G1switch	85.6 (6.42)
FOS	v-fos FBJ murine osteosarcoma viral oncogene homolog	78.3 (6.29)
SORBS1^a^	sorbin and SH3 domain containing 1	72.0 (6.17)
PLIN^ab^	Perilipin	68.1 (6.09)
PRKAR2B^a^	PRKAR2B a protein kinase, cAMP-dependent, regulatory, type IIb	53.1 (5.73)
CHRDL1^a^	chordin-like 1	52.0 (5.70)
APOD^a^	apolipoprotein D	49.9 (5.64)
PPP1R1A	protein phosphatase 1, regulatory (inhibitor) subunit 1A	41.4 (5.37)
GHR	growth hormone receptor	41.4 (5.37)
AOC3^ab^	amine oxidase, copper containing 3 (vascular adhesion protein 1)	40.8 (5.35)
CLEC3B	C-type lectin domain family 3, member B	38.1 (5.25)
DPT^a^	dermatopontin	37.0 (5.21)
NTRK2	neurotrophic tyrosine kinase, receptor, type 2	36.5 (5.19)
PALMD	palmdelphin	34.1 (5.09)
ACACB	acetyl-Coenzyme A carboxylase beta	32.2 (5.01)
LEP^a^	leptin	28.8 (4.85)
VWF	von Willebrand factor	28.1 (4.81)
TIMP4^b^	TIMP metallopeptidase inhibitor 4	26.7 (4.74)
COL11A1^ab^	collagen, type XI, alpha 1	−11.7 (−3.55)

The table shows 25 genes (represented by 28 probe sets) whose expression continuously increases or decreases from less differentiated to more differentiated samples as ranked in Fig. 6. The genes are ordered according to their fold change in expression between mesenchymal stem cells and normal fat cells. These genes are related to adipogenesis. About 64% of those genes have previously been reported in [6] and [27] (marked with a and b, respectively).

also reported by ^a^ Matushansky et al. [6] and ^b^ Sekiya et al. [31].

### Comparison of tree reconstruction methods to other algorithms

**Figure 7:**
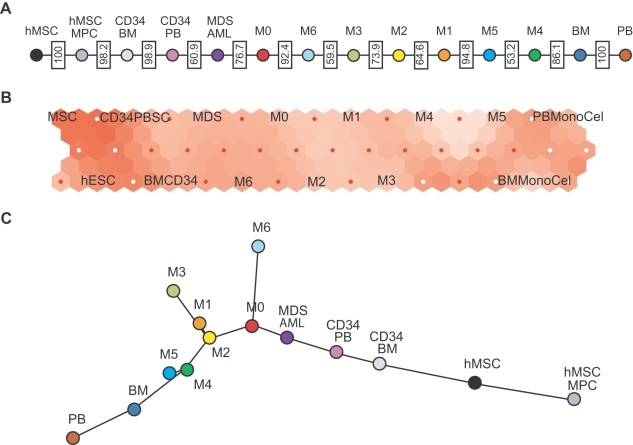
Alternate distance based methods applied to acute myeloid leukemia (AML) data. (**a**) The figure shows the results of a simple algorithm that sorts the AML subtypes by their distance to hESC. The algorithm uses the same distances as the ones for the phylogenetic tree shown in Fig. 2. (**b**) Self-Organizing Maps. The AML subtypes are arranged on a hexagonal grid of 15×3 nodes. These nodes are visualized by the small red or white dots. The colors visualize the difference of neighboring nodes. For example, the light nodes surrounding M4 and M5 show that these subtypes are similar. MSC and CD34+ peripheral blood, however, show very different expression patterns despite the fact that they are ordered close together on the map. (**c**) Minimum Spanning Tree (MST) calculation of the Pearson correlation matrix of the AML dataset.

We compare the results obtained from phylogenetic tree reconstruction algorithms with other methods of data clustering and organization such as a simple greedy algorithm (in which subtypes are linearly ordered by their distance from hESC), self-organizing maps (SOMs), and minimum spanning trees (MSTs) (see the Methods section for details of the algorithms). When applying the greedy algorithm to our AML dataset, we find similar results to those produced by the tree reconstruction analysis (Fig. 7a). Although the correspondence between the results of this method and the reconstructed phylogenetic tree is very good, the former only contain information of a linear organization, as opposed to the richer information that can be extracted from the tree topology and branch lengths. An example of a self-organizing map (SOM) algorithm applied to the AML dataset is shown in Fig. 7b. Subtypes that are known to be similar are mapped close together on the grid – e.g. human embryonic stem cells (hESC), mesenchymal stem cells (MSC), and samples with markers of poor differentiation (BMCD34 and CD34PB). Unfortunately, the overall organization of a SOM strongly depends on the shape and size of the grid, making it difficult to interpret the results in a robust and useful way for our purposes. Finally, we calculate a minimum spanning tree (MST) for the AML dataset (Fig. 7c). This algorithm accurately reproduces the reconstructed tree found with our original method, with the exception of mesenchymal stem cells being placed at the edge of the tree (instead of embryonic stem cells).

### Systematic analysis of methods and parameters

We compare the different methodologies implemented in our algorithm for each step of the analysis in order to identify those methods and parameters that perform well in the analysis of our datasets. We apply our algorithm to all datasets using all combinations of the following methods and parameters: for finding differentially expressed genes: ANOVA, Kruskal-Wallis (KW) and Welch approximation (Welch); two methodologies for p-value correction: Benjamini-Hochberg (BH) and Holm; two p-value cutoffs: 0.01 and 0.05; five tree reconstruction and clustering algorithms: Weighted Least Squares (WLS), Minimum Evolution (ME), Neighbor-Joining (NJ), FastME, and Average Linkage (UPGMA); and two distance measures: Pearson correlation and Euclidean distance. The results of these analyses are shown in Figs. S1, S2, S3, S4. The topologies found among the different combinations of parameters show that WLS, Pearson correlation, and BH with a cutoff value of 0.01 perform accurately in accordance with the AML (Fig. S1), breast cancer (Fig. S2), and liposarcoma datasets (Fig. S3).

Note that two main assumptions of the UPGMA algorithm are not fulfilled by cancer subtype data, namely: all species originate from a common ancestor and they all have evolved at the same pace. This issue explains why this method fails to reconstruct the right tree topologies; for example, in all sarcoma UPGMA topologies (trees 1 and 4 of Fig. S4), some liposarcoma subtypes branch together with leiomyosarcoma, which is thought to arise from smooth muscle tissue.

It has been shown in previous studies that, in general, WLS performs better than NJ when trees have long external or internal branches (e.g. [33]). Note also that the use of Euclidean distance leads to less robust results than the use of Pearson correlation when trees with long branches are considered. For example, when the Euclidean distance method is applied to the liposarcoma data, the dedifferentiated and pleomorphic subtypes cluster together with the well-differentiated subtype and normal fat (Topology 3 of Fig. S3). The effect of long branches on the Euclidean distance method becomes even more pronounced when analyzing the sarcoma data (Fig. S4); in this case, the least common topologies are observed only when the Euclidean distance method is used. If distant subgroups (i.e. hMSC and hMSC MPC) are removed from the analysis, then most parameter combinations including the Euclidean distance method favor topology 5. This topology was previously only observed with the Pearson correlation distance (see Table in Fig. S4, left).

We do not observe a significant influence of the choice of the method on the identification of differentially expressed genes. More important for our data is the choice of the p-value cutoff. For the sarcoma data, conservative p-value cutoffs favor topology 3 while parameter combinations with Benjamini-Hochberg adjusted p-values seem to favor topology 5 (Fig. S4). The results of our study suggest that BH with a cutoff of 0.01 is a good compromise, but we recommend investigating the effects of using different cutoff values.

In general, all tree reconstruction methods are very fast, especially since the number of different tumor subtypes in our analysis is typically limited. So it is possible to test many parameters in a reasonable time and we recommend doing so.

## Discussion

We have presented a rational methodology to investigate the dissimilarity between cancer subtypes and stem cells. Our approach uses gene expression data of tumor samples which have been classified into histological subtypes as well as expression data of an ‘evolutionary outgroup’ such as embryonic stem cells, tissue-specific stem cells, and/or fully differentiated normal cells. The data of tumor subtypes is used to identify the genes that are differentially expressed among the subtypes, and those genes, together with data of the outgroup, allows construction of a phylogeny of cancers. Our algorithm estimates the statistical significance of the tree branches by bootstrapping, a repeated tree construction using a varying number of randomly chosen genes. The distance between the branching points of the tumor subtypes and the stem cells specifies their dissimilarity, which is caused in part by differences in maturity, and ranks the subtypes according to increasing differentiation. This ranking is then used to identify genes whose expression continuously changes depending on the degree of maturation.

Our methodology is validated by being able to correctly reproduce experimental results concerning the relationship in differentiation status of liposarcoma, breast cancer and AML subtypes [2], [6], [18] and concerning genes related to adipocytic differentiation [6], [31]. Our method is useful for identifying genes that are overexpressed in some tumor subtypes (Fig. 6c). For instance, genes whose expression is increased in a particular tumor type but not in normal tissue-specific stem cells and differentiated cells may represent candidates for targeted therapy, possibly with lessened side effects. Interestingly, some of the genes found to be differentially expressed in only one or a few liposarcoma subtypes can be targeted by currently available drugs. It will be an important next step to test those genes for a causal role in tumorigenesis.

In recent years, bioinformatic tools have been widely used to analyze the vast amount of data produced experimentally. In analyses of microarray data, simple algorithms for phylogenetic tree reconstruction, such as Average linkage (UPGMA) [34], produce rooted bifurcating trees and are routinely applied to visualize similarities in gene expression. The most prominent example for this type of analysis are heatmaps, a graphical representation of the clustered expression matrix where colors represent the measured gene intensities; a dendrogram is often added which shows the bifurcating tree best describing the differences in gene expression [35]. Another important application of such algorithms is the clustering of tumor samples for improving or discovering subtype classifications (e.g. [36]). Other more sophisticated tree reconstruction algorithms are only rarely applied to expression data [22], [37]–[42]. The ‘molecular clock’ assumption of UPGMA (specifying that changes occur at a constant rate, [43]) renders this algorithm inappropriate for our investigation. Other algorithms such as Maximum Parsimony, Neighbor-Joining (NJ) [44], or Least-Squares [45] enable us to root the tree and to estimate the differentiation status of tumor subtypes by a simple comparison of the lengths between the root of the tree and the branching points of the leaves. We do not use character-based methods such as Maximum Parsimony due to the necessity of artificially discretizing the continuous values of gene expression intensities.

The estimation of evolutionary distances between tumors from gene expression data is hindered by the fact that small differences in the biology of tumors may cause large differences in gene expression. Examples of such situations are given by genes which trigger the expression of cascades of other genes [40] and mutational events affecting the expression of several genes [46]. In a recent paper [46], Park et al. proposed the use of correction methods that estimate *mutational* distances from the observed expression distances. This approach represents an interesting new avenue to further explore in future work.

The phylogeny of tumor subtypes identified by our methodology cannot be used to reconstruct the evolutionary history of a single tumor sample. The fact that dedifferentiated liposarcomas, for example, branch earlier than well-differentiated liposarcomas is not to be taken as evidence that one subtype evolved into the other. Rather, it specifies the dissimilarity of the bulk of tumor cells between cancer subtypes from stem cells at the time of observation. Similarly, our methodology cannot be used to identify the cell of origin of a tumor type. Both the position of a subtype in a differentiation-based phylogeny and the similarity of a subtype to an *in vitro* differentiation time course provide information about the bulk of tumor cells only; to determine whether these cells are produced from tumor stem cells which arose from cells of similar, earlier or more complete differentiation stages is outside the scope of this approach. Furthermore, the ability of a phylogenetic tree to reconstruct evolutionary trajectories when applied to genetic data rests on the assumption that the genetic material records the evolutionary history of the system. In the case of phenotypic information such as gene expression data, this assumption does not hold, and hence any information about the origin of the investigated cancer subtypes cannot be obtained.

The generality of our approach and the extensive availability of high-quality input datasets (e.g. GEO) makes this methodology a unique tool to investigate differentiation-related genes and the relationship in maturity of cancer subtypes. The use of data from patient samples reduces the problems encountered with *in vitro* studies regarding the reproducibility of the results in other systems and their significance to *in vivo* situations.

## Methods

### Dataset compilation

We use gene expression data of sarcoma samples from Singer et al. [27] and Barrentina et al [30]. The gene expression was measured on Affymetrix U133a oligonucleotide arrays. The classification in [27] was performed using unsupervised hierarchical clustering and an SVM-based supervised classification method. To root the tree, we use expression data of 17 normal fat samples from the same study as well as expression data of 3 human embryonic stem cell lines (hESCs) and 3 hESC derived mesenchymal precursor lines (downloaded from NCBI Geo [47] accession number GSE7332 [48]). We use gene expression data of AML [47] patient samples available within GEO (accession numbers GSE1159, GSE9476 [49], GSE1729 [50], and GSE12417 [51]). The breast cancer dataset is also compiled from Microarray data published in GEO with dataset numbers GSE7390 [16], GSE2990 [15], GSE3494 [17], and GSE9574 [23]. A problem of micrarray meta-analyses is that the different dataset sources may introduce a bias. We therefore applied hierachical clustering to the compiled breast cancer dataset and did not observe a clustering according to the sources.

### Statistical methods and analysis

#### Data preprocessing

The CEL files are normalized and summarized with the *rma* function of *Bioconductor 2.2* [52]–[54]. For the phylogenetic tree construction and mainly as a strategy to remove potential noise from the data, we only consider genes that show significant differences in their expression profiles when comparing tumor subtypes. These differentially expressed genes are determined with a one-way ANOVA. In addition, our R scripts support as alternative methods for finding differentially expressed genes the Welch approximation (R function *oneway.test(…, var.equal = FALSE)*) [55] and the Kruskal-Wallis test (*kruskal.test()*) [56]. As default cutoff we choose Benjamini-Hochberg corrected p-values [20] of 0.01. To obtain a differentiation baseline, we include expression data of normal fully differentiated tissue and, as an outgroup for the phylogenetic tree construction, the expression profile of tissue-specific stem cells. Pairwise distances of the cancer subtypes and baseline samples are computed with the Pearson Correlation Distance (*d* = 1-*p*) or the Euclidean Distance of the average group intensities.

#### Phylogenetic tree reconstruction methods

The phylogenetic trees are reconstructed with several distance-based methods. The *fitch* program includes the implementations of the Weighted Least Squares (WLS) [45] and Minimum Evolution (ME) [57] methods, *neighbor* provides the Neighbor-Joining (NJ) [44] and UPGMA algorithms [34]. Both programs are available in the *Phylip* package version 3.67 [58]. WLS and ME are methods designed to find the tree topology that fits the distance matrix best by optimization. The difference between these two algorithms is the optimization criterion. WLS minimizes the sum of squares error of the distances in the tree (*d^T^*) compared to the ones in the distance matrix (δ):
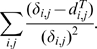
The denominator thus *weighs* the deviations of *δ* from *d^T^* for distantly related species less. As we often have very distant *in vitro* outgroups in the data, this is an important reason for us to choose WLS as the default tree reconstruction method. ME uses the same criterion to fit branch lengths to a given tree topology as WLS, but returns the topology with the smallest sum of branch lengths, not the one with the smallest sum of squares error. Another related method is Balanced Minimum Evolution (BME), implemented in the FastME program [39]. Both WLS and BME have shown good performance on microarray data [38]. FastME is orders of magnitude faster than the *Phylip* implementation of WLS and thus suitable for very large datasets. NJ is another computationally very efficient distance-based tree reconstruction method and also popular because of its accuracy (e.g. [59]). UPGMA [34] is a hierarchical clustering algorithm that works in a ‘bottom-up’ way: at the beginning, all elements form individual clusters which are consecutively combined until all elements are contained in only a single cluster. In each iteration, the pair with the smallest distance is combined into a higher-level cluster and the distance matrix is updated by calculating the distances to the newly formed cluster. The strength of the algorithm is twofold: it is computationally very efficient and it does not depend on the *a priori* selection of the number of clusters, in contrast to the *k*-means or SOMs algorithms.

#### Bootstrapping procedure

To assess the statistical significance of the phylogeny, the reconstruction is repeated 10,000 times with random subsets of the differentially expressed genes. We draw the bootstrap sample size *n* from the discrete uniform distribution on the interval [50, *N*], where *N* is the total number of differentially expressed genes. Then *n* genes are sampled with replacement from the set of these *N* genes. We further bootstrap the tumor samples to incorporate the uncertainty of tumor classification. Therefore we sample for each tumor subtype *n_i_* experiments with replacement from the set of the *n_i_* experiments of this subtype. Once a consensus tree is determined, it is rooted and visualized with *Dendroscope* version 2.2.2 [60].

#### Profile clustering

The phylogenetic tree explicitly specifies the differentiation order in the internal branch nodes. We then use the order of samples determined by the tree to calculate expression profile clusters with *mfuzz* [61], a fuzzy c-means R package commonly used for clustering profiles of time series. This algorithm is similar to the *k*-means algorithm and returns the probabilities that a gene belongs to particular expression profile cluster. As in the *k*-means algorithm, the number of expression profile clusters has to be set in advance and was set to 20 for the clustering of liposarcoma expression profiles in Fig. 4.

### Comparison of our methodology to other clustering and dimension-reduction methods

#### Greedy ordering of subtypes

We use a naïve greedy algorithm in which subtypes are linearly ordered by their distance from hESC. The distance calculation and the bootstrapping are equivalent to the ones used by the phylogenetic tree reconstruction. Bootstrap values can be interpreted exactly as in the phylogenetic trees, i.e. peripheral blood samples are positioned farthest from hESC in all replicates (bootstrap value of 100%), while M5 is located closest to peripheral blood mononuclear cells in 82% of the replicates.

#### Self-Organizing Maps (SOMs) [62]

SOMs are a type of unsupervised clustering algorithms that map high-dimensional data into a 2-dimensional grid – typically hexagonal or rectangular. The number of nodes in the grid must be set in advance, similarly to the *k*-means algorithm where the number of clusters is a predefined variable. The algorithm results in a two-dimensional map where similar data points tend to cluster together. SOMs are commonly applied to microarray data to cluster both genes [63] and tumors [64]. We calculate SOMs with the original implementation in the *SOM_PAK* version 3.1 [65] with the averaged group intensities of all differentially expressed genes (ANOVA FDR 0.01). We set the topology to hexagonal and choose the ‘bubble’ neighboring kernel.

#### Minimum Spanning Trees (MSTs)

MSTs are a well-established concept in graph theory. A spanning tree of a connected weighted graph *G* is an acyclic connected subgraph of *G* with the same set of vertices as *G*. A distance matrix can now be interpreted as a complete graph in which the edge weights correspond to the distances. The MST is the spanning tree that connects all vertices of *G* with the smallest sum of edge weights. MSTs have been shown to be useful for clustering and classification of microarray data [66]. For the MST calculation we use the *spantree* function of R, which is an implementation of Prim's algorithm [67]. We apply this function to the Pearson distance matrix calculated again with all differentially expressed genes. A major disadvantage of this method is the lack of an established algorithm to find consensus MSTs for the resulting trees after bootstrapping, in contrast to phylogenetic trees where the availability of a wide range of methods and implementations makes it easy to summarize bootstrap results (e.g. [68], [69]). Furthermore, there are no ancestral states (inner nodes) in an MST, as opposed to phylogenetic trees where subtypes are leaves in the tree and other nodes are created as ancestral states.

### Availability

The R code and the compiled AML dataset are available from the authors upon request. A user-friendly GUI that supports most of the methods described in this paper is available as Plugin for MAYDAY [70].

## Supporting Information

Figure S1 Acute myeloid leukemia (AML) consensus tree topologies. The trees in this figure correspond to all 38 topologies of the bootstrap consensus trees of 120 different combinations of parameters (see Methods for details). Topology 7 is the one produced by our default parameter combination: ANOVA, BH, p-value cutoff 0.1 and WLS with Pearson correlation distance (Fig. 2). The table shows the parameters that return the given topologies. Topology 7, for instance, is only observed with the Pearson correlation distance, and topologies 8, 13, 17, 25, 31, 33 and 37 are reconstructed only by the UPGMA algorithm.
(0.64 MB EPS)

Figure S2 Breast cancer consensus tree topologies. The figure shows the ten different consensus trees we obtain by applying 60 different parameter combinations to the breast cancer dataset. The tree topologies resulting from the UPGMA algorithm and the Euclidean distance (2, 4, 5, 7, 8, 9) are unable to identify the right order of subtypes. The remaining parameters do not have such a strong effect when using this dataset (Topologies 1, 3, 6, 10): EP+G1 is always the most differentiated subtype and EP-G3 is always closest to stem cells. Topologies 1 and 6 are favored by NJ and FastME, while topology 3 is favored by Fitch and ME.
(0.62 MB EPS)

Figure S3 Liposarcoma consensus tree topologies. This figure shows the four different topologies of the consensus trees of 120 different parameter combinations. Topology 2 corresponds to our default parameter combination (Fig. 4a). The table shows that this topology is not observed when the Euclidean distance is chosen. UPGMA also never returns this topology. The numbers show that other parameters have little or no effect on the analysis of this data.
(0.31 MB EPS)

Figure S4 Sarcoma consensus tree topologies. This figure shows all eight different consensus trees we obtain by applying 120 different parameter combinations to the sarcoma data. Topology 5 is the one produced by our default parameter combination (see Fig. 5) while topology 3 is the same but with the order of DD and PL switched. The numbers in the table show again that these topologies are never observed when the Euclidean distance is chosen. FastME and NJ favor topology 3 while WLS and ME favor topology 5. Conservative p-value cutoffs such as Holm 0.01 seem to favor topology 3. Again, UPGMA never produces these topologies. The choice of the distance measure is the biggest determinant of the tree topology.
(0.57 MB EPS)
